# Reducing inverse quantization numbers in intra frame for video transcoding architectures

**DOI:** 10.1371/journal.pone.0215131

**Published:** 2019-05-08

**Authors:** Ming-Te Wu

**Affiliations:** Department of Information Technology, Kao Yuan University, Kaohsiung, Taiwan; Nanjing University of Information Science and Technology, CHINA

## Abstract

In this study, a complexity-quality analysis with transcoding architectures is proposed for reducing inverse quantization numbers. This architecture is different from conventional transcoding scheme, which neglects the relationship between previous and current quantizer step size. However, the proposed transcoding architecture depends on the modulus of the ratio of the current and previous quantization parameter. By analyzing the quantized area of the previous and current quantization parameter, we concluded the part of undoing first inverse quantization, to reduce computing complexity. From computer simulation, we verify the merits of the proposed scheme over the conventional transcoding approaches, in terms of achieving better performance based on the computing complexity and objective (e.g., the peak signal-to-noise ratio) analysis.

## Introduction

Transcoding is very important in multimedia application. When we would like to share good videos with friends especially, it is a very well way by internet transmission. Limited to internet bandwidth, if we want to deliver video bitstreams, the bit-rate conversion problem we will face. On the other hand, it is also a transcoding problem. Generally, transcoding can be interpreted as the operation of converting a video from one format into another format [1]. For example, an original video is encoded in an MPEG-2 format at 5.3Mb/s, the temporal rate is 30 f/s, and the input resolution is 720×480. Then the original video is transcoded to an MPEG-4 format at 128Kb/s, the temporal rate is 10f/s, and the output resolution is 352×240 [2]. However, the meaning of transcoding is not only an operation of format-conversion but also it can share popular video-audio to another people through the internet or satellite media. This will propagate information unlimitedly.

There are many transcoding application schemes, including the bit-rate reduction, spatial resolution reduction, temporal resolution (skipped frame) reduction, and error-resilience transcoding [3]. The straightforward method is transcoding in pixel-domain [4] which is a direct cascade decoder and encoder approach. That is, the incoming bitstreams are first decoded in the pixel domain, and then the decoded video frames are re-encoded at the bit-rate which client’s demands. But the drawbacks of this scheme are high computing complexity and too much memory cost. To reduce the complexity, Youn etc. [5] proposed information reusing method which is a skill that motion vectors from the input bitstreams after decoding can be reused to reduce the computing complexity of transcoder. A distributed video transcoding scheme that uses dependency among a group of pictures by preparing video blocks of variable size was proposed to reduce the bitrate and transcoding time for fast delivery of a video to end users [6]. Van etc. [7] developed several schemes to reduce the computation of closed-loop translating for high-efficiency video coding. A high bit rate input bitstream is decoded and the recovered sequence is then re-encoded at a lower bit rate. A new fast transcoding algorithm to make full use of the prior knowledge of the influence of video brightness on transcoding modes was proposed [8]. It used the information available from previously decoded MBs and YUV difference to decide which modes can be overpassed with little loss to the rate-distortion performance. Jokhio etc. [9] present prediction-based dynamic resource allocation and deallocation algorithms to a dynamically scalable cluster of video transcoding servers. A Hadoop-based distributed video transcoding method that transcodes various video codec formats into the MPEG-4 video format was proposed [10]. Improvements in quality and speed are achieved by adopting the Xuggler Java library for transcoding based on open source.

Early research almost used the re-quantization methods on the transcoder to reduce the complexity [11], [12]. But this kind of method often causes degraded performance by high reduction ratio required by the re-quantization method. Therefore, the frame-skipping technique was introduced [13], [14]. This technique can significantly reduce the bit-rate to match internet bandwidth demands. However, the drawback of this technique is increasing more computing complexity at reconstructing the skipped frame procedure. Using the coefficients of discrete cosine transform and predicted modes, Lin [15] proposed a transcoding method by reducing largest coding unit and early ending. To improve high efficient video coding, Wan [16] developed a transcoder with boosted bit-rate by exploiting the architecture of cascaded pixel-domain. Kim [17] employed quadtree framework with different downscaled resolutions to boost the high efficient video coding transcoder.

**Fig 1:**
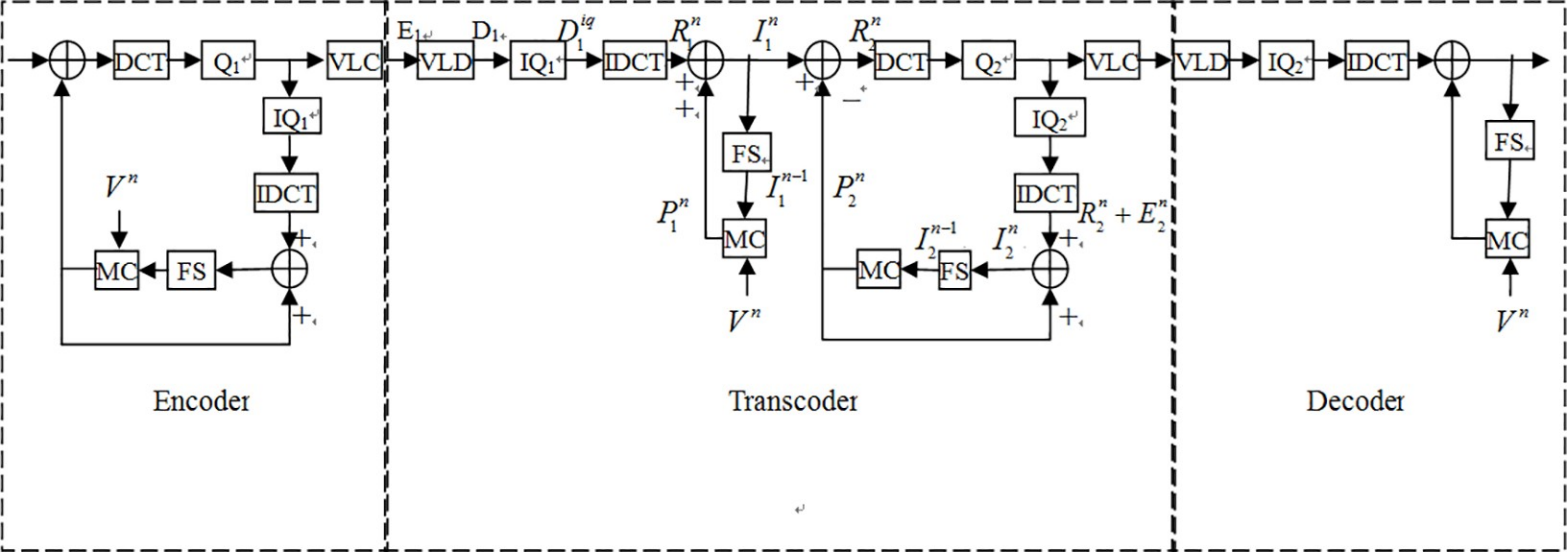
Encoder-transcoder-decoder architecture.

For details, Fig 1 describes an encoder-transcoder-decoder common architecture. Table 1 is a nomenclature list of abbreviations. When we would like to transcode one original format bitstreams which were encoded data, the E_1_ in Fig 1, to new format bitstreams, the first step is decoding E_1_ data to D_1._ Then, by inverse first quantization (IQ_1_), the D_1_ transform to $D_{1}^{iq}$ data. Following, using inverse discrete cosine transform (IDCT) method transforms the $D_{1}^{iq}$ in the frequency domain to the $R_{1}^{n}$ in the spatial domain and adds the motion compensation vector from motion compensator (MC) to combine a compensated bitstreams $I_{1}^{n}$. Then $I_{1}^{n}$ data subtract the motion compensation vector from MC and after DCT and second quantization (Q_2_), the bitstreams could be encoded and be delivered to the decoder.

Because the transcoder architecture of Fig 1 is computing complexly, Vetro etc. [3] also proposed a bit-rate reduction method to reduce the bit-rate and maintain the original frames performance. However, the reason that reduced computing complexity by bit-rate reduction method is discarding a few high-frequency data. But the tradeoff is to degrade the performance. Besides, Vetro etc. proposed another scheme, names spatial resolution reduction. This scheme used down-sampling four macroblocks (MBs) to one MB, the associated motion vectors have to be mapped, that is, a reduction factor of two in both the horizontal and vertical resolution. In this case, each motion vector is mapped from 16×16 MB in the original resolution to an 8×8 block in the reduced resolution MB with appropriate scaling by two. Though the down conversion scheme can reduce the number of motion vectors, oppositely it needs to calculate the new motion vectors. The most important point is that this scheme will cause worse distortion because of the error between new motion vectors and original motion vectors.

In this paper, a complexity-quality analysis for transcoding architectures of reducing inverse quantization numbers is proposed. This architecture is different from conventional transcoding scheme, which neglects the relation between first and second quantizer step size. However, our proposed transcoding architecture depends on the modulus of the ratio of the second quantization and first quantization. By analyzing the quantized area of first quantization and second quantization, we conclude the part of undoing first inverse quantization, to reduce computing complexity. From computer simulation, we verify the merits of the proposed scheme over the conventional approaches, in terms of achieving superior performance based on the computing complexity and objective analysis.

For discussion, this paper is organized as follows; in Section 2, conventional transcoder architecture is first introduced and then the novel modified transcoder architecture is proposed. In Sec. 3, the results of the simulation are provided that confirm and demonstrate the effectiveness of the algorithm, in comparison to the conventional transcoder scheme, in terms of computing complexity reduction. Finally, conclusions are presented in Sec. 4.

## Modified transcoder architecture

In this section, we proposed a new architecture which according to the modulus that the quantized step size at transcoder divides the quantized step size at the encoder. We designed several different transcoding processes according to the different modulus of quantization ratio cases. This benefits that transcoding will spend the least computing complexity and maintain the same performance. We will do the computing complexity reduction analysis by PSNR measure objectively and vision measure subjectively in Sec.3.

### Conventional transcoder architecture

**Fig 2:**
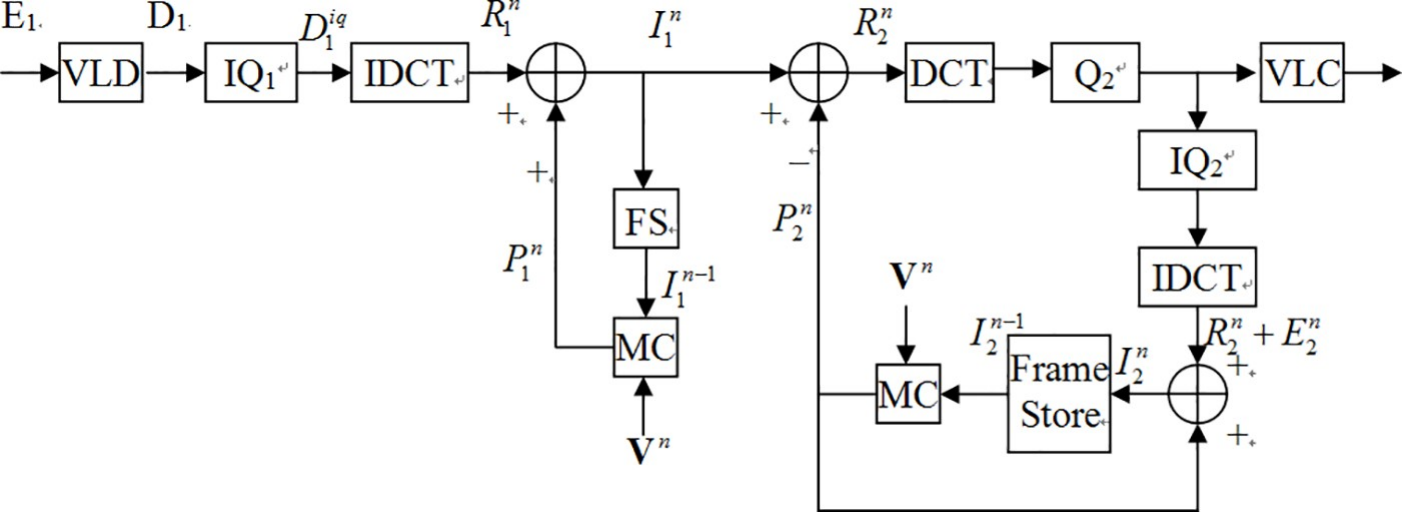
CPDT transcoder transcoding architecture.

Fig 2 describes a pixel-domain transcoding architecture, named cascaded pixel-domain transcoder (CPDT) [18].

Because the predicted frame $P_{1}^{n}$ is the composition in which the sum of the spatial position vector of the (*n*-1)-th original picture $I_{1}^{n-1}$ and the spatial position vector of the motion compensation vector **V**^*n*^. Hence, $P_{1}^{n}$ can be indicated as

$$P_{1}^{n}(x)=I_{1}^{n-1}(x+V^{n}(x))$$ (1)

The decoded frame $I_{1}^{n}(x)$ can be yielded by the residual frame $R_{1}^{n}$ which was inverse discrete cosine transform (IDCT) adding to the predicted frame $P_{1}^{n}$, that is,

$$I_{1}^{n}(x)=R_{1}^{n}(x)+P_{1}^{n}(x)$$ (2)

Substituting (1) into (2), (2) can be rewritten as

$$I_{1}^{n}(x)=R_{1}^{n}(x)+I_{1}^{n-1}(x+V^{n}(x))$$ (3)

From Fig 2, we can see that the residual frame $R_{2}^{n}$ is the one which the decoded picture $I_{1}^{n}(x)$ subtract the predicted frame $P_{2}^{n}$. So $R_{2}^{n}$ can be represented as

$$R_{2}^{n}(x)=I_{1}^{n}(x)-P_{2}^{n}(x)$$ (4)

Furthermore, after DCT, Q_2_, IQ_2_, and IDCT, the residual frame $R_{2}^{n}$ must introduce a quantized error $E_{2}^{n}$. Hence, the frame $I_{2}^{n}$ can be denoted as

$$I_{2}^{n}(x)=P_{2}^{n}(x)+R_{2}^{n}(x)+E_{2}^{n}(x)$$

Take (4) into account, we could rewrite $I_{2}^{n}$ as

$$\begin{array}{l} I_{2}^{n}(x)=I_{1}^{n}(x)-R_{2}^{n}(x)+R_{2}^{n}(x)+E_{2}^{n}(x)\\ \;=I_{1}^{n}(x)+E_{2}^{n}(x) \end{array}$$ (5)

Besides, the predicted frame $P_{2}^{n}$ is the composition in which the sum of the spatial position vector of the (*n*-1)-th original picture $I_{2}^{n-1}$ and the spatial position vector of the motion compensation vector **V**^*n*^. Therefore, $P_{2}^{n}$ can be indicated as

$$P_{2}^{n}(x)=I_{2}^{n-1}(x+V^{n}(x))$$ (6)

Substituting (5) into (6), (6) can be rewritten as

$$P_{2}^{n}(x)=I_{1}^{n-1}(x+V^{n}(x))+E_{2}^{n-1}(x+V^{n}(x))$$ (7)

Substituting (7) into (4), the relation between the residual picture $R_{2}^{n}$ and the decoded frame $I_{1}^{n}(x)$ is following,

$$R_{2}^{n}(x)=I_{1}^{n}(x)-I_{1}^{n-1}(x+V^{n}(x))-E_{2}^{n-1}(x+V^{n}(x))$$ (8)

To get the correlation between the residual frame $R_{2}^{n}$ and the residual frame $R_{1}^{n}$, we substituted (3) into (8) and yielded

$$R_{2}^{n}(x)=R_{1}^{n}(x)-E_{2}^{n-1}(x+V^{n}(x))$$ (9)

**Fig 3:**
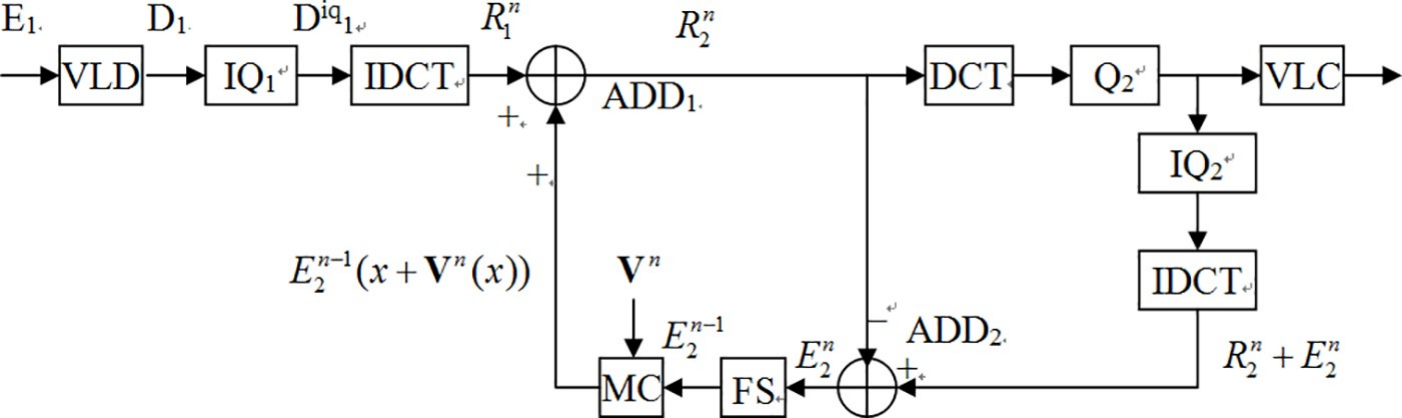
Simplified architecture using the correlation between $R_{2}^{n}$ and $R_{1}^{n}$.

Hence, we simplified Fig 2 to Fig 3. In fact, because DCT and IDCT are all linear operations, the result in Fig 3 would not be changed despite performing adding arithmetic or (I)DCT prior. Therefore, we could move the IDCT operator behind adder ADD1 in Fig 3.

As mentioned above, according to the linear property of DCT and IDCT, we could move the IDCT block from left end to right end of Position X and the IDCT and DCT can cancel each other.

By linear property of IDCT, we move IDCT block below Position X to the left of the ADD_2_ adder.

**Fig 4:**
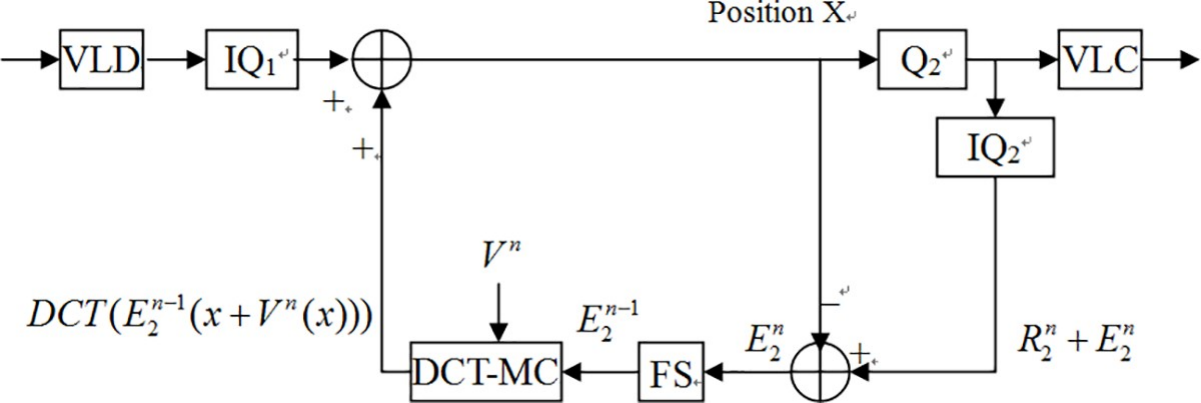
SDDT transcoder.

**Fig 5:**
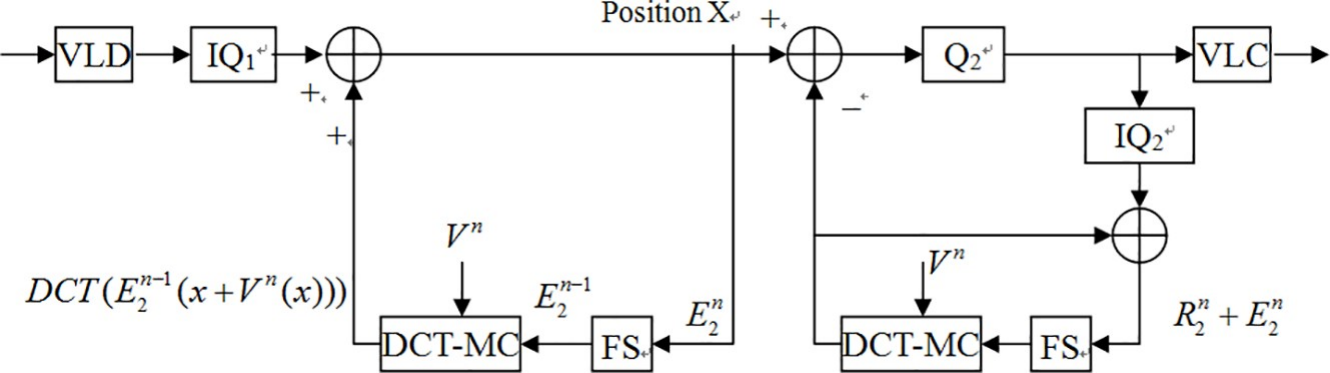
CDDT transcoder.

Because the incoming bitstreams from IQ_1_ are frequency domain coefficients, if the coefficients in the close-loop also are frequency domain coefficients, then it is not necessary to perform IDCT. To simply the (I)DCT blocks, DCT domain transcoding was introduced [18]. Hence, if we converse motion compensate (MC) in the spatial domain to DCT-MC in the frequency domain [19], then we can take away DCT and IDCT and the transcoder can be simplified as Fig 4 which named as simplified DCT-domain transcoder (SDDT) [19][4]. In fact, though SDDT architecture reduces the number of (I)DCT, it increases the computing complexity which the process of MC converting to DCT-MC introduces. Another drawback of SDDT architecture is that it can only be employed at which the encoder and decoder have the same spatial/temporal resolution. In addition, the output video and input video need use the same motion vectors and encoding modes. Thus, the cascaded DCT-domain transcoder (CDDT) [20] in Fig 5 was introduced. However, though the CDDT improves the usable limits, oppositely it increases the complexity of DCT-MC and frame store blocks. Follows, we proposed a novel method to reduce the complexity and still maintain the PSNR.

### The Auto-selective transcoder architecture

In this section, we would like to propose a new modified version of transcoding architecture, with auto-selective architecture capability, for computational complexity reduction of bitstreams, during video transcoding processes. We employ the modulus of the ratio of the first quantizer step size and second quantizer step size to design different scheme. That is, the transcoding architecture relies on the modulus that the quantized step size at transcoder divides the quantized step size at the encoder, i.e., mod(*Q*_2_/*Q*_1_).

**Fig 6:**
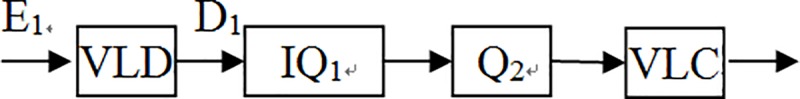
I-pictures transcoding architecture.

Whatever the group-of-pictures (GOP) structure of input bitstreams is, the I-pictures are the major elements which spend the most memory. The others P-pictures or B-pictures need only store the motion vector (MV) which the motion estimator (ME) in encoder estimated. So we will reduce the computing complexity to I-pictures below. When the input bitstreams are Intra pictures (I-pictures), these I-pictures need not perform a motion estimate. Hence, we could simply Fig 4 to Fig 6.

**Fig 7:**
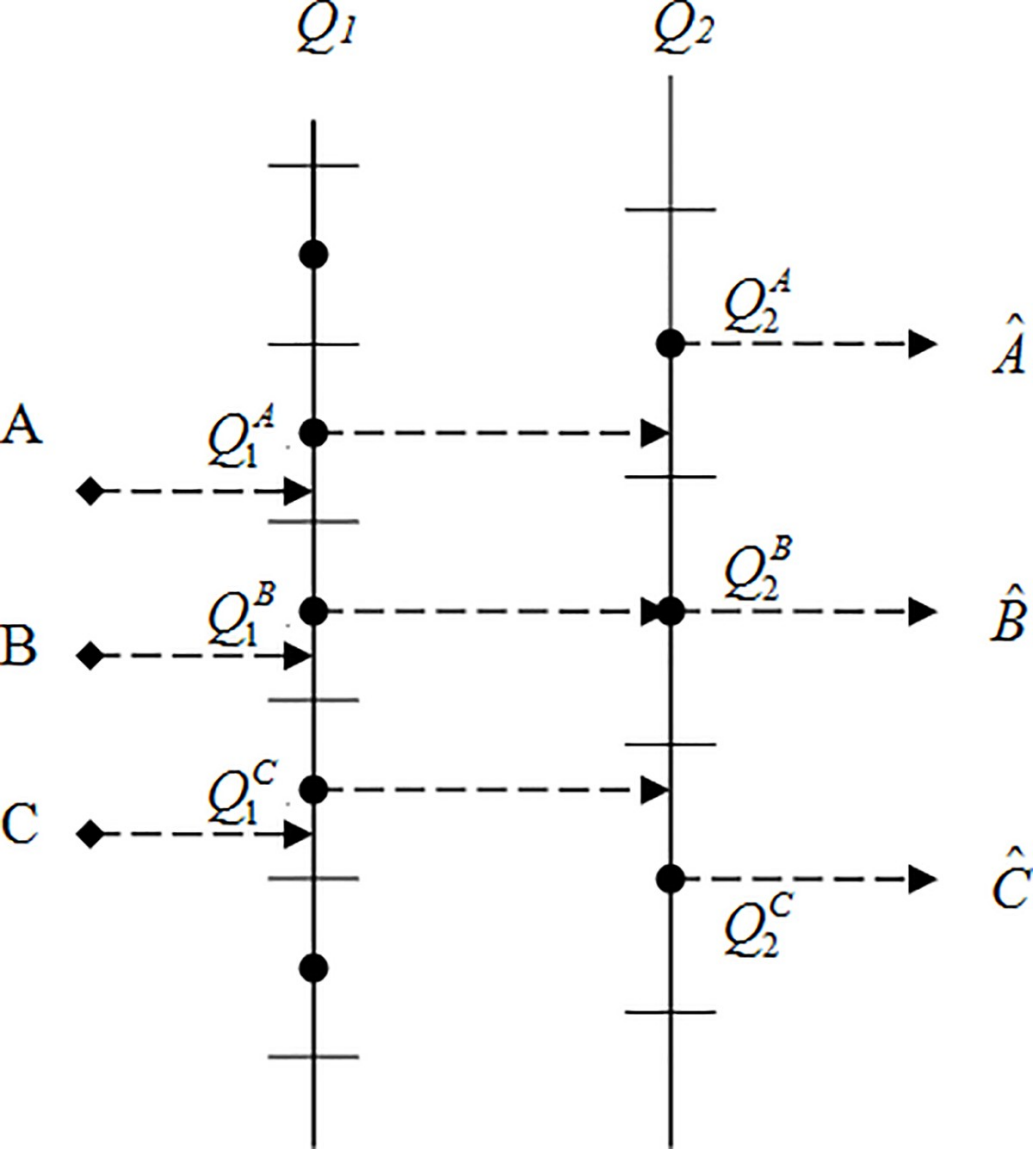
Quantization in pixel domain.

In P-pictures/B-pictures, because P-pictures/B-pictures are the composition of motion compensates vectors and the residual frame, the P-pictures need consider Fig 4 architecture. Whatever the incoming bitstreams are, they all need to consider the re-quantization problem. This is because that if the value of mod(*Q*_2_/*Q*_1_) is not an integer when performing second quantization, it will cause performance error. This error may be stated as following from Fig 7. In Fig 7, point A is first quantized to $Q_{1}^{A}$ and denoted $Q_{1}(A)=Q_{1}^{A}$. The $Q_{1}^{A}$ is then second quantized to $Q_{2}^{A}$ and yield the point $\hat{A}$ which is stated

$$\hat{A}=Q_{2}(Q_{1}^{A})=Q_{2}^{A}$$ (10)

Similarly, point B is first quantized to $Q_{1}^{B}$, and is expressed as $Q_{1}(B)=Q_{1}^{B}$. Then $Q_{1}^{B}$ is second quantized to $Q_{2}^{B}$ and yield the point $\hat{B}$ which is indicated $\hat{B}=Q_{2}(Q_{1}^{B})=Q_{2}^{B}$.

It is worth mentioning that if point A is first quantized and then directly second quantized, it will get the result of (10). However, if point A is first quantized and perform inverse first quantizing and then continue second quantizing, the result is different from (10) and can be indicated as

$$\hat{B}=Q_{2}(A)=Q_{2}^{B}$$ (11)

**Fig 8:**
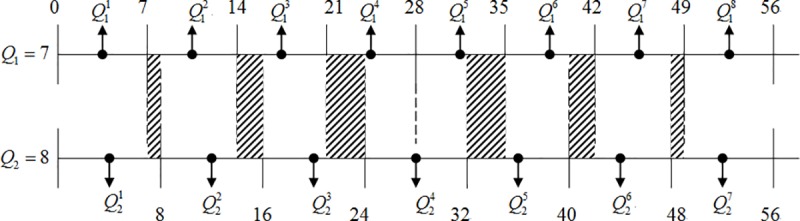
Quantization of mod(Q_2_/Q_1_) in pixel domain.

Clearly, it will introduce so-called quantized error. In this paper, we classified different schemes using the values of the mod(*Q*_2_/*Q*_1_). When *Q*_1_ = 7 and *Q*_2_ = 8, the shadow region in Fig 8 is the part of the quantized error. We can see that the shadow regions are far smaller than no shadow ones. On the other hand, the quantized error regions are far smaller than direct cascaded quantization regions. Hence we have an idea that the input bitstreams possess auto-selective probability of performing inverse quantization, that is, inversely quantize only on those bitstreams of shadow regions. The other no shadow regions can directly perform cascade quantization. This method benefits reducing computing complexity which every block pixels need to inverse quantize *IQ*_1_ and accompanying second quantize *Q*_2_.

Theoretically, if we know the pixel value of bitstreams and the pixel value of input point A which is between 7 and 8 in Fig 8, then the first quantized point A will get the value of $Q_{1}^{2}$. Not performing inverse quantization *IQ*_1_ but directly second quantizing *Q*_2_, it will then yield the value of $Q_{2}^{2}$. In fact, if point A first inverse quantized *IQ*_1_ and then did second quantization *Q*_2_, it will get the value of $Q_{2}^{1}$. Unfortunately, the input bitstreams which we received at the transcoder are quantized value *Q*_1_, but not original frame pixel values. Hence, we can not perform second quantization using the original frame pixels. If we do not want to perform inverse quantization and directly second quantize, we can see from Fig 8 that only 0~7 and 49~56 can directly second quantize *Q*_2_. The other $Q_{1}^{2}\sim Q_{1}^{7}$ all need to do inverse quantization. Despite a few pixels needing to inverse quantize, we still can reduce 2/8 computing complexity which needs to inverse quantize in Fig 8.

If we set *Q*_1_ = *m*, we can summarize a general expression as follow,

$$\left\{ \begin{array}{l} Q_{2}(Q_{1}^{m})=Q_{2}^{m},\;if\;\mathrm{mod}(\frac{Q_{2}}{Q_{1}})=0\\ Q_{2}(Q_{1}^{n})=Q_{2}^{n}\;and\;Q_{2}(Q_{1}^{m+\mathrm{mod}(\frac{Q_{2}}{Q_{1}})})=Q_{2}^{m},\;if\;\mathrm{mod}(\frac{Q_{2}}{Q_{1}})\neq 0\;and\;n=1\\ Q_{2}(Q_{1}^{n})=Q_{2}(IQ_{1}^{n}(Q_{1}^{n})),\;if\;n\in [2,m+\mathrm{mod}(\frac{Q_{2}}{Q_{1}})-1] \end{array}\right.$$ (12)

**Table 1:** Different quantization modes of switch SW of the proposed transcoder in I-pictures.

Quantization mode	SW Position
**Non IQ**	A
**IQ**	B

**Fig 9:**
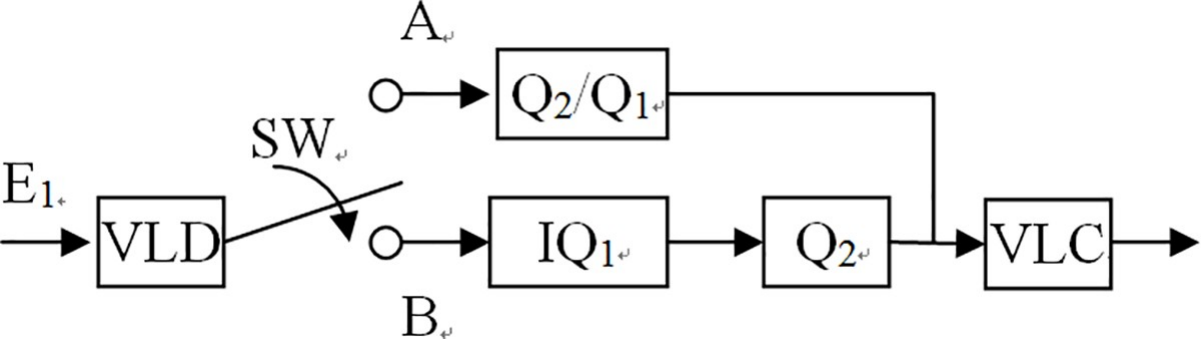
Modified I-pictures transcoding architecture.

According to the energy concentration property of DCT, the input bitstreams picture pixels which are DCT and quantized, the most part values of them are small and concentrated in $Q_{1}^{1}$ exclude the DC value. Therefore we can perform the first proposed method on AC values and DC values still do inverse first quantization *IQ*_1_ and accompanying second quantization *Q*_2_. Thus, we can modify Fig 6 to Fig 9. Table 1 describe different quantization modes of switch SW of Fig 9. When the SW position at A, the bitstreams do not perform inverse quantization. When the SW position at B, the bitstreams perform IQ_1_. However, we only use software to design the switch instead of hardware architecture. It would not spend any hardware cost. Thus, our proposed method can be employed to not only reduce more computing complexity but also to maintain good performance.

## Experimental results

**Fig 10:**
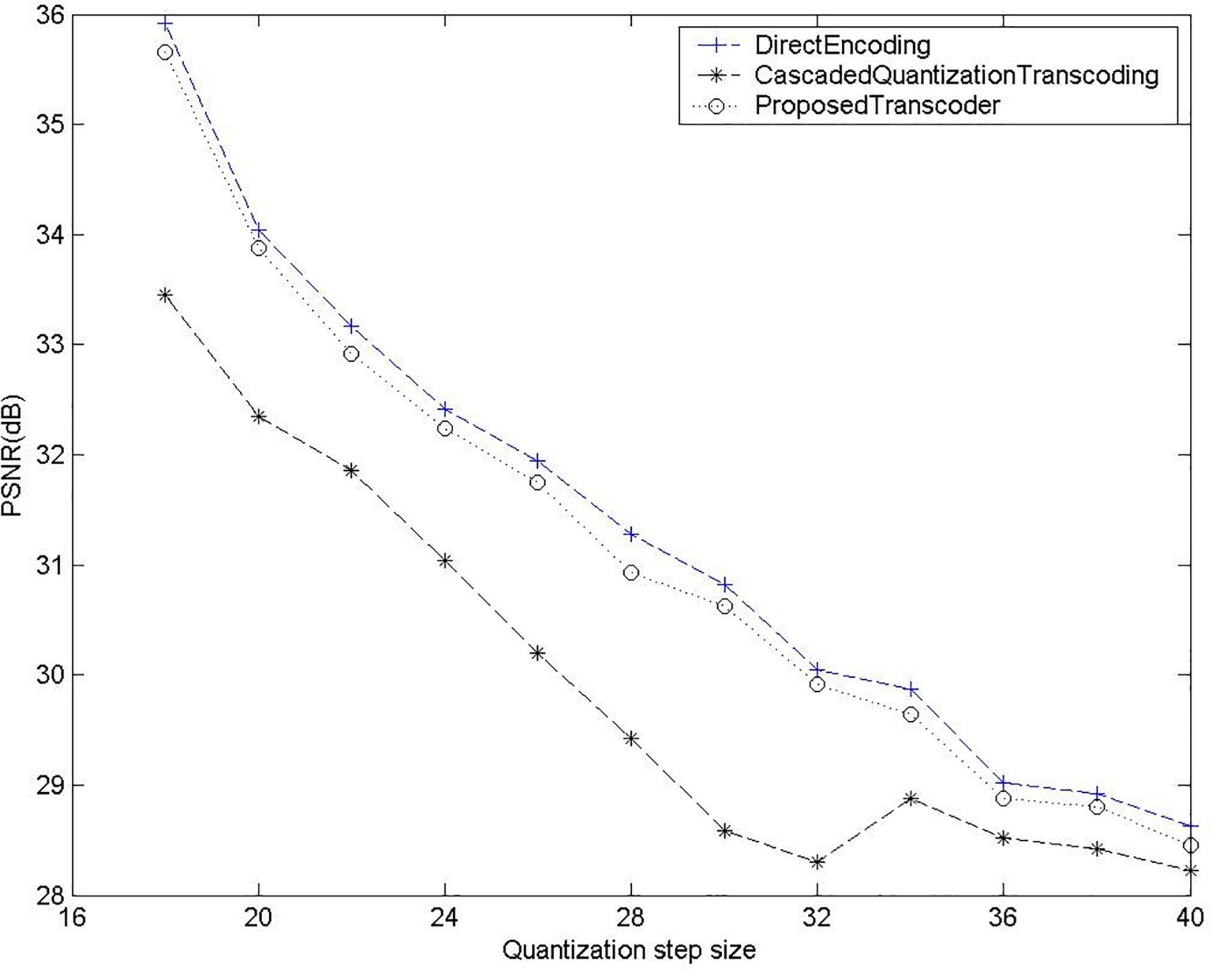
Intra-frame transcoding of *Flower Garden* encoded Q_1_ = 16, transcode different Q_2_.

**Fig 11:**
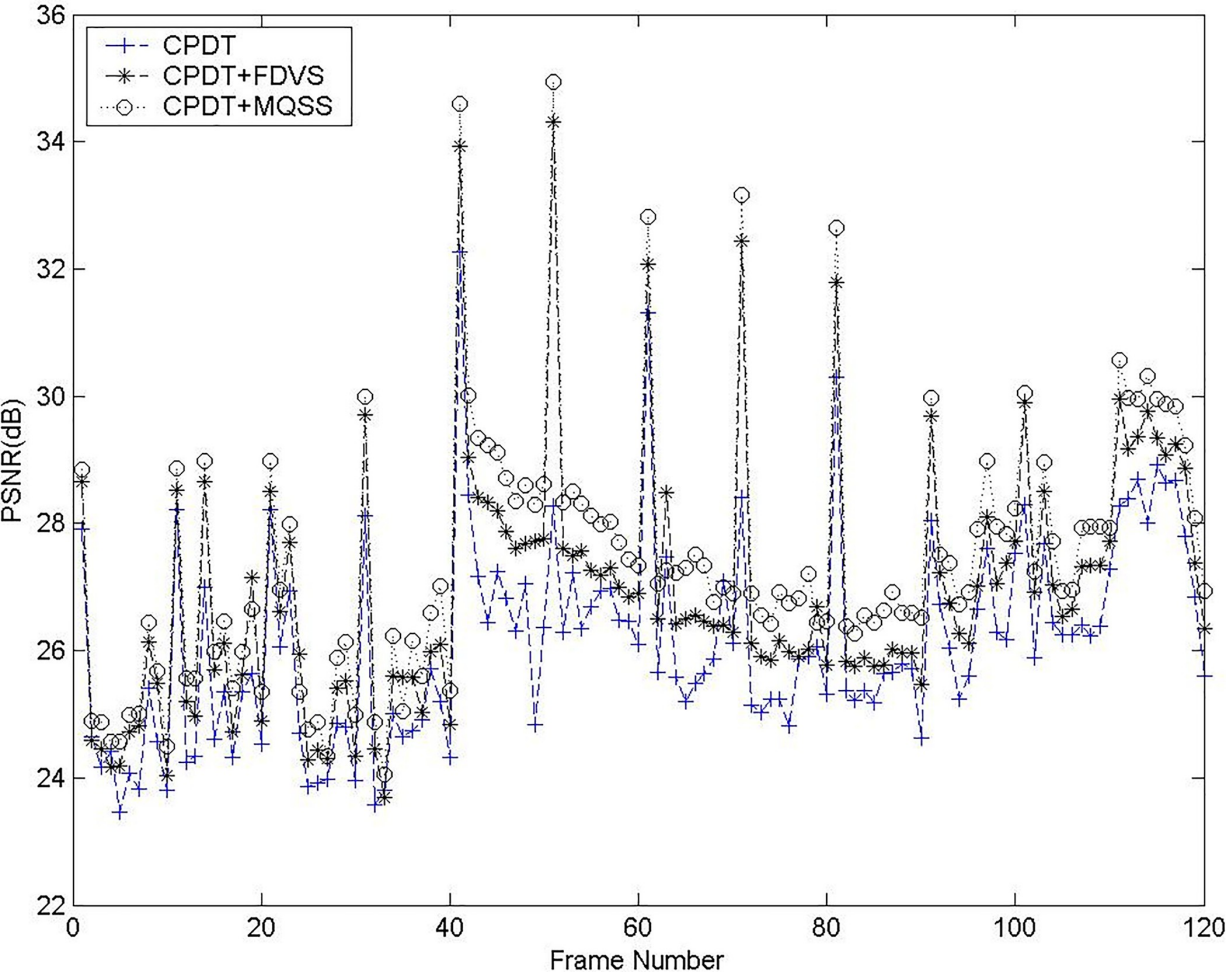
PSNR comparison for Flower Garden.

In this section, the superiority, in terms of good visual quality and good peak signal noise ratio (PSNR), of the proposed scheme is verified using computer simulation. For comparison, the 352×288 CIF and 3840×2160 4-k ultra-HD test sequences, viz., *Foreman*, *Susie*, *Mobile & Calendar*, Cactus and *Flower Garden* are chosen for the data compression process and adopted as simulation sequences. The experiments are performed on a Pentium-IV 1.6GHz PC. Several experiments are made in MPEG II. In fact, the proposed method can be implemented in any coding standard because all transcoding architecture need to process the I-picture of decoding/encoding. From Table 2, we can see that our proposed method is faster than CPDT about 21.3fps, SDDT about 5fps, CDDT about 14.2fps in IPPP… case for the *Foreman* sequences. In IBBP… case, our proposed method is faster than CPDT about 14.2fps, SDDT about 4.2fps, CDDT about 11.6fps for the *Foreman* sequences. Besides, we can see that our proposed method is faster than CPDT about 21.5fps, SDDT about 5.1fps, CDDT about 12.4fps in IPPP… case for the *Mobile & Calendar* sequences. In IBBP… case, our proposed method is faster than CPDT about 15.1fps, SDDT about 4.3fps, CDDT about 11.1fps for the *Mobile & Calendar* sequences. Table 3 shows our proposed method has better PSNR than CPDT approach about 0.12~0.42 dB and CPDT+FDVS [14] scheme about 0.05~0.28 dB. In Fig 10, the PSNR of our proposed method was about 0.1–0.3 dB less than that of the direct encoding approach but perform better than cascaded quantization transcoding for the *Flower Garden* sequences. However, the complexity in I-picture transcoding of the proposed scheme was reduced by about 20%, while maintaining good visual performance. Additionally, Fig 11 displays that the proposed system has better objective performance than the other methods. In addition, 4-k ultra-HD video clips are test in Table 4.

**Table 2:** The comparison of the proposed MQSS scheme using SDDT with CPDT, SDDT, CDDT, in terms of fps, for the *Foreman*, *Mobile & Calendar* and *Susie* CIF sequences which are encoded at QP = 7, and then transcoded at QP = 15.

Sequences (352 ×288)	Foreman		Mobile & Calendar		Susie	
	IPPP…	IBBP…	IPPP…	IBBP…	IPPP…	IBBP…
**CPDT**	7.0 fps	7.1 fps	6.6 fps	6.7 fps	7.9	8.0
**SDDT**	23.2 fps	17.1 fps	23.0 fps	17.5 fps	26.2	19.3
**CDDT**	14.1 fps	9.7 fps	15.7 fps	10.7 fps	15.9	10.9
**SDDT+MQSS**	28.3 fps	21.3 fps	28.1 fps	21.8 fps	32.0	24.1

**Table 3:** Average PSNR comparison using MPEG-2 as a front encoder, for the *Susie*, *Mobile & Calendar* and *Foreman* sequences which are encoded at QP = 7, and then transcoded at QP = 15.

Sequences	Method	Average PSNR
Input bitrate is 3Mbps with 30 frames/s		
**Susie**	CPDT	40.78
	CPDT+FDVS	40.92
	CPDT+MQSS	41.2
**Mobile & Calendar**	CPDT	26.39
	CPDT+FDVS	26.43
	CPDT+MQSS	26.55
**Foreman**	CPDT	30.03
	CPDT+FDVS	30.1
	CPDT+MQSS	30.15

**Table 4:** The comparison of the proposed MQSS scheme using SDDT with CPDT, SDDT, CDDT, in terms of fps, for the *Foreman*, *Mobil & Calendar*, *susie* and *Cactus* 4-k ultra-HD sequences which are encoded at QP = 7, and then transcoded at QP = 15.

Sequences (3840 ×2160)	Foreman		Mobile&Calendar		Susie		Cactus	
	IPPP…	IBBP…	IPPP…	IBBP…	IPPP…	IBBP…	IPPP…	IBBP…
**CPDT**	0.170fps	0.172fps	0.162fps	0.164fps	0.192fps	0.196fps	0.174fps	0.176fps
**SDDT**	0.566fps	0.418fps	0.562fps	0.428fps	0.640fps	0.532fps	0.580fps	0.428fps
**CDDT**	0.344fps	0.236fps	0.384fps	0.262fps	0.388fps	0.266fps	0.352fps	0.242fps
**SDDT+MQSS**	0.692fps	0.522fps	0.686fps	0.534fps	0.782fps	0.590fps	0.710fps	0.534fps

This study developed novel modified transcoding architecture, with auto-selective architecture capability, which reduces the computational complexity of video transcoding. Experimental results show that the proposed method can yield better vision and PSNR performance than other approaches.

## Conclusions

In this paper, we have proposed a new modified version of transcoding architecture, with auto-selective architecture capability, for computational complexity reduction of bitstreams, during video transcoding processes. Experimental results show that our method can obtain good vision and PSNR performance in comparison with other approaches.

## Appendix

### A Proof That Proposed Video Transcoding Architectures

The all re-quantization possibilities we summarized as follows:

Case 1: *Q*_1_ = *Q*_2_ = 7

That is, $\mathrm{mod}(\frac{Q_{2}}{Q_{1}})=0$, we can directly quantize by *Q*_2_ and need not to perform inverse first quantization *IQ*_1_. Therefore we can reduce 100% computing complexity which needs to inverse quantize and can be expressed as

$$Q_{2}(Q_{1}^{n})=Q_{2}^{n},\;if\;\mathrm{mod}(\frac{Q_{2}}{Q_{1}})=0\;and\;n\in [1,8]$$ (A.1)

Case 2: *Q*_1_ = 7 and *Q*_2_ = 8

When $\mathrm{mod}(\frac{Q_{2}}{Q_{1}})=1$, two quantized regions $Q_{1}^{1}$ and $Q_{1}^{8}$ can be directly quantized by *Q*_2_, $Q_{2}(Q_{1}^{1})=Q_{2}^{1}$ and $Q_{2}(Q_{1}^{8})=Q_{2}^{7}$, and $Q_{1}^{2}\sim Q_{1}^{7}$ need to perform inverse quantization. So we can reduce at least $\frac{2}{8}$ (25%) computing complexity which needs to inverse first quantize *IQ*_1_ and be indicated as

$$\left\{ \begin{array}{l} Q_{2}(Q_{1}^{1})=Q_{2}^{1}\;and\;Q_{2}(Q_{1}^{8})=Q_{2}^{7}\;if\;\mathrm{mod}(\frac{Q_{2}}{Q_{1}})=1\\ Q_{2}(Q_{1}^{n})=Q_{2}(IQ_{1}^{n}(Q_{1}^{n}))\;if\;n\in [2,7] \end{array}\right.$$ (A.2)

Case 3: *Q*_1_ = 7 and *Q*_2_ = 9

When $\mathrm{mod}(\frac{Q_{2}}{Q_{1}})=2$, two quantized regions $Q_{1}^{1}$ and $Q_{1}^{9}$ can be directly quantized by *Q*_2_, $Q_{2}(Q_{1}^{1})=Q_{2}^{1}$ and $Q_{2}(Q_{1}^{9})=Q_{2}^{8}$, and $Q_{1}^{2}\sim Q_{1}^{8}$ need to perform inverse quantization. So we can reduce $\frac{2}{9}$ (22.2%) computing complexity which needs to inverse first quantize *IQ*_1_ and be stated

$$\left\{ \begin{array}{l} Q_{2}(Q_{1}^{1})=Q_{2}^{1}\;and\;Q_{2}(Q_{1}^{9})=Q_{2}^{7}\;if\;\mathrm{mod}(\frac{Q_{2}}{Q_{1}})=2\\ Q_{2}(Q_{1}^{n})=Q_{2}(IQ_{1}^{n}(Q_{1}^{n}))\;if\;n\in [2,8] \end{array}\right.$$ (A.3)

Case 4: *Q*_1_ = 7 and *Q*_2_ = 10

In the same way as (13), when $\mathrm{mod}(\frac{Q_{2}}{Q_{1}})=3$, we can reduce $\frac{2}{10}$ (20%) computing complexity which needs to inverse first quantize *IQ*_1_ and be stated

$$\left\{ \begin{array}{l} Q_{2}(Q_{1}^{1})=Q_{2}^{1}\;and\;Q_{2}(Q_{1}^{10})=Q_{2}^{7}\;if\;\mathrm{mod}(\frac{Q_{2}}{Q_{1}})=3\\ Q_{2}(Q_{1}^{n})=Q_{2}(IQ_{1}^{n}(Q_{1}^{n}))\;if\;n\in [2,9] \end{array}\right.$$ (A.4)

Case 5: *Q*_1_ = 7 and *Q*_2_ = 11

When $\mathrm{mod}(\frac{Q_{2}}{Q_{1}})=4$, we can reduce $\frac{2}{11}$ (18.2%) computing complexity which needs to inverse first quantize *IQ*_1_ and be stated

$$\left\{ \begin{array}{l} Q_{2}(Q_{1}^{1})=Q_{2}^{1}\;and\;Q_{2}(Q_{1}^{11})=Q_{2}^{7}\;if\;\mathrm{mod}(\frac{Q_{2}}{Q_{1}})=4\\ Q_{2}(Q_{1}^{n})=Q_{2}(IQ_{1}^{n}(Q_{1}^{n}))\;if\;n\in [2,10] \end{array}\right.$$ (A.5)

Case 6: *Q*_1_ = 7 and *Q*_2_ = 12

When $\mathrm{mod}(\frac{Q_{2}}{Q_{1}})=5$, we can reduce $\frac{2}{12}$ (16.7%) computing complexity which needs to inverse first quantize *IQ*_1_ and be stated

$$\left\{ \begin{array}{l} Q_{2}(Q_{1}^{1})=Q_{2}^{1}\;and\;Q_{2}(Q_{1}^{12})=Q_{2}^{7}\;if\;\mathrm{mod}(\frac{Q_{2}}{Q_{1}})=5\\ Q_{2}(Q_{1}^{n})=Q_{2}(IQ_{1}^{n}(Q_{1}^{n}))\;if\;n\in [2,11] \end{array}\right.$$ (A.6)

Case 7: *Q*_1_ = 7 and *Q*_2_ = 13

When $\mathrm{mod}(\frac{Q_{2}}{Q_{1}})=6$, we can reduce $\frac{2}{13}$ (15.4%) computing complexity which needs to inverse first quantize *IQ*_1_ and be stated

$$\left\{ \begin{array}{l} Q_{2}(Q_{1}^{1})=Q_{2}^{1}\;and\;Q_{2}(Q_{1}^{13})=Q_{2}^{7}\;if\mathrm{mod}(\frac{Q_{2}}{Q_{1}})=6\\ Q_{2}(Q_{1}^{n})=Q_{2}(IQ_{1}^{n}(Q_{1}^{n}))\;if\;n\in [2,12] \end{array}\right.$$ (A.7)

## Nomenclature

VLD: Variable length decoding
IQ1: Inverse first quantization
IDCT: Inverse discrete cosine transform
FS: Frame store
MC: Motion compensation
DCT: discrete cosine transform
Q_2_: Quantizer in transcoder
IQ_2_: Inverse second quantization
VLC: Variable length encoding
*V^n^*: The nth motion vector
E_1_: Encoded data
D_1_: Decoded data
$D_{1}^{iq}$: Inverse quantized data
$R_{1}^{n}$: Residual data in decoder end of transcoder
$I_{1}^{n}$: The nth intraframe data in decoder end of transcoder
$I_{1}^{n-1}$: The (n-1)th intraframe data in decoder end of transcoder
$P_{1}^{n}$: Predictive data in decoder end of transcoder
$R_{2}^{n}$: Residual data in encoder end of transcoder
$P_{2}^{n}$: Predictive data in encoder end of transcoder
$I_{2}^{n}$: The nth intraframe data in encoder end of transcoder
$I_{2}^{n-1}$: The (n-1)th intraframe data in encoder end of transcoder
$E_{2}^{n}$: Quantized error in encoder end of transcoder
